# N6‐methyladenosine demethylase FTO suppresses clear cell renal cell carcinoma through a novel FTO‐PGC‐1α signalling axis

**DOI:** 10.1111/jcmm.14128

**Published:** 2019-01-16

**Authors:** Changshui Zhuang, Chengle Zhuang, Xiaomin Luo, Xinbo Huang, Lv Yao, Jianfa Li, Yawen Li, Tiefu Xiong, Jing Ye, Fangting Zhang, Yaoting Gui

**Affiliations:** ^1^ The Guangdong and Shenzhen Key Laboratory of Male Reproductive Medicine and Genetics Peking University Shenzhen Hospital, Institute of Urology of Shenzhen PKU‐HKUST Medical Center Shenzhen P.R. China; ^2^ Assisted Reproduction Unit, Department of Obstetrics and Gynecology Sir Run Run Shaw Hospital, Zhejiang University School of Medicine Hangzhou P.R. China

**Keywords:** ccRCC, FTO, m6A, mitochondria, oxidative stress, PGC‐1α

## Abstract

The abundant and reversible N6‐methyladenosine (m6A) RNA modification and its modulators have important roles in regulating various gene expression and biological processes. Here, we demonstrate that fat mass and obesity associated (FTO), as an m6A demethylase, plays a critical anti‐tumorigenic role in clear cell renal cell carcinoma (ccRCC). FTO is suppressed in ccRCC tissue. The low expression of FTO in human ccRCC correlates with increased tumour severity and poor patient survival. The Von Hippel‐Lindau‐deficient cells expressing FTO restores mitochondrial activity, induces oxidative stress and ROS production and shows impaired tumour growth, through increasing expression of PGC‐1α by reducing m6A levels in its mRNA transcripts. Our work demonstrates the functional importance of the m6A methylation and its modulator, and uncovers a critical FTO‐PGC‐1α axis for developing effective therapeutic strategies in the treatment of ccRCC.

## 1 INTRODUCTION

Clear cell renal cell carcinoma (ccRCC), derived from renal epithelium, makes up nearly 75% of all kidney cancer.^1^ Most patients who present with metastatic disease have a dismal prognosis since ccRCC exhibits few or no symptoms early and advances in therapy have been limited. Constitutive activation hypoxia inducible factor (HIF) signalling,^2^ “Warburg effect”^3^ and low mitochondrial function^4^ indicate that these profound shifts in cellular metabolism take place during ccRCC tumorigenesis. Metabolic reprogramming enables kidney cancer cells to rapidly proliferate, survive in conditions of nutrient depletion and hypoxia and evade the immune system,^5^ thus describing this tumour type as a “metabolic disease”.^2^, ^5^

Von Hippel‐Lindau (VHL) mutations or deletions are the most frequent genetic alterations in ccRCC.^6^, ^7^, ^8^ The Vhl gene, encoding an E3 ubiquitin ligase, is essential for oxygen‐dependent degradation of HIFα family.^9^ However, in hypoxia, HIFα degradation is inhibited, leading to HIFα stabilization, increased nuclear localization of HIFα and transcription of various target genes, including the vascular endothelial growth factor. Consequently, VHL loss of function in ccRCC leads to constitutive activation of HIF‐α, which promotes tumorigenesis through transcriptional activation of genes mediating angiogenesis,^10^ anti‐apoptosis^11^ and metabolism.^12^ Constitutive activation of HIF transcription factors have been placed a high value on metabolic reprogramming in ccRCC. HIFα drive a gene expression program that increases glycolytic activity while inhibiting mitochondrial function.^13^, ^14^ In ccRCC, low mitochondrial content is associated with tumour aggressiveness, suggested that inhibition of mitochondrial function may play a key role in ccRCC progression.^4^ However, the mechanism underlying reduced mitochondrial content in ccRCC, and its subsequent events, remains less understood.

The PPARg coactivators (PGC) are a family of transcriptional coactivators (consisting of PGC‐1α, PGC‐1β and PRC) that mediate mitochondrial biogenesis and oxidative phosphorylation. But PGC‐1α, as a central regulator of mitochondrial function, cannot be compensated for by the other family members.^15^, ^16^ Accumulating evidence indicates PGC‐1α play a dualistic role in cancer, with reports of tumour suppression and pro‐tumorigenic effects of PGC‐1α expression in variant cancer types.^17^, ^18^, ^19^ A convincingly better understanding of the role of PGC‐1α in variant tumour types will be highly significant in exploring whether this target will be amenable as anticancer agents in ccRCCs.

N6‐Methyladenosine (m6A) is the most abundant internal post‐transcriptional modification in mRNA occurring particularly at the beginning of the 3′‐UTR near the stop codon, usually embedded within the consensus motif RR(m6)ACH (R=G or A,H=U, A or C).^20^, ^21^, ^22^, ^23^ This reversible modification, installed by a methyltransferase complex consisting of the proteins methyltransferase‐like 3 (METTL3), METTL14 and Wilms tumour 1 associated protein (WTAP) identified as m6A “writers”, is “erased” by fat mass and obesity‐associated protein (FTO) and AlkB homolog 5 (ALKBH5).^24^, ^25^ Recent studies have shown that m6A modification in mRNAs plays critical roles in mRNA splicing and translation efficiency.^26^, ^27^, ^28^, ^29^ However, m6A modification has been most strongly related to increased mRNA instability.^30^ Other groups reported that suppressor of cytokine signalling 2(SOCS2) mRNA degradation mediated by METTL3 promotes liver cancer progression,^31^ that ALKBH5 maintains cancer stem‐like cells phenotype by sustaining NANOG and FOXM1 mRNA stability^32^, ^33^ and that FTO‐regulated ADAM19 promotes the tumorigenesis of brain tumour,^34^ suggesting the biological significance of the mRNA m6A methylation and its modulators in cancer development. However, the impact of FTO, especially as an RNA demethylase, in mitochondrial biogenesis, oxidative stress and ccRCC progression remain elusive. Importantly, recent reports suggest that FTO regulate mitochondria content through mediating mitochondrial fusion, fission and biogenesis‐associated genes expression as a N6‐methyladenosine RNA demethylase.^35^, ^36^ To the point, we aim to explore the biological function of FTO in the post‐transcriptional modification of mitochondrial biogenesis and its subsequent influence in ccRCC and also explore the underlying molecular mechanism through identifying its key mRNA targets.

## 2 MATERIALS AND METHODS

### 2.1 Human samples, tissue microarray and cell lines

**Figure 1:**
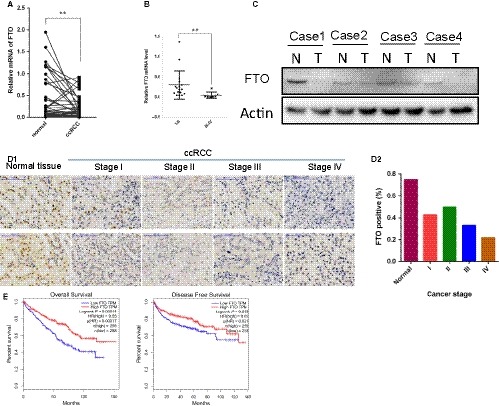
FTO is down‐regulated in clear cell renal cell carcinoma (ccRCC) and its expression is progressively lost during kidney cancer progression. (A) qRT‐PCR analysis of FTO mRNA expression in matched normal kidney and ccRCC tumour (n = 35) samples. (B) FTO mRNA expression in ccRCC tumours was analysed according to tumour stage. (C) FTO protein level in four paired human ccRCC biopsies and matched normal tissue analysed by Western blot. (D1) IHC analysis of FTO on a tissue array of ccRCC patients using the Allred score. Twenty‐five paired samples were analysed, and eight representative samples with loss of expression in tumour are shown. Scale bars, 100 mm. (D2) The IHC signals were scored as 0, 1, 2 and 3; a score ≥1 indicated FTO positive detection. (E) Kaplan‐Meier curves for overall survival and disease free survival of ccRCC patients grouped on the basis of PGC‐1 expression. Error bars represent the mean ± SD, **P < 0.01

All ccRCC samples from Figure 1A‐C and Figure 6A were collected from patients who had ccRCC resection performed at Peking University Shenzhen Hospital. The samples were used for subsequent RNA extraction or immunohistochemistry (IHC). All human materials were obtained with informed consent. The ccRCC tissue microarray (#HKid‐CRC060CS‐01) was purchased from Shanghai Outdo Biotech CO. Ltd (Shanghai, China) and IHC stained for FTO. Human embryonic kidney (HEK) 293T cells and human kidney cancer cell lines, 786‐O and 769‐P were purchased from American Type Culture Collection (ATCC) and cultured as suggested by ATCC's guidelines. All tumour collection and analysis were approved by the Peking University Shenzhen Hospital Institutional Review Board with informed consent.

### 2.2 Plasmid, RNA interference and lentiviral production

FTO expression and PGC‐1α expression plasmids (pCMV3‐FTO‐FLAG and pCMV3‐PGC1α‐FLAG) were purchased from Sino Biological (Beijing, China). To generate FTO (H231A and D233A), site‐directed mutagenesis was performed using the QuickChange Site‐Directed mutagenesis kit (Agilent, Santa Clara, CA, USA) and the resulting plasmids were sequence verified. For plasmid transfection, cells were transfected with plasmid using Lipofectamine TM 3000 (ThermoFisher, Waltham, MA, USA, #L3000015) as suggested instructions; siRNAs against FTO and PGC‐1α were designed and synthesized by GenePharma (Shanghai, China). For RNA interference, cells were transfected with 50 nmol/L siRNA or a control siRNA using Lipofectamine RNAiMAX (ThermoFisher, #13778150) per the manufacturer's instructions. Total RNA was isolated 48 hours later for real‐time PCR analysis. The siRNA sequences are listed in Table S1.

Lentivirus for FTO, FTO‐mut as well as control were packaged with psPAX2, pMD2G (Addgene, Watertown, MA, USA) and pCDH‐puro into HEK‐293T cells. To establish stable cell lines, the concentrated lentivirus were directly added into cancer cells and incubated at 37°C for 48 hours before they were washed out with phosphate‐buffered saline (PBS). Finally, cells were selected with 2.5 mg/mL puromycin for 4 days.

### 2.3 RT‐qPCR and gene‐specific m6A qPCR

Total RNA was isolated from cultured cells using TRIzol (Invitrogen, Waltham, MA, USA) according to the manufacturer's instructions. The cDNA synthesis was performed using the Transcript First‐Strand cDNA Synthesis SuperMix Kit (TransGen Biotech, Beijing, China). Quantitative real‐time PCR (qRT‐PCR) was performed with SYBR Premix Ex Taq II (Tli RNaseH Plus) (Takara, Dalian, China, #RR820). Relative genes expression was tested by 2^∆∆Ct^ normalized to GAPDH; gene ‐specific m6A qPCR were conducted as described previously.^32^, ^37^ Relative m6A‐genes expression was tested by ^2∆∆Ct^ normalized to hypoxanthine guanine phosphoribosyl transferase (HPRT) according to the reason that HPRT mRNA did not have m6A peaks from the m6A seq data.^30^ Real‐time PCR was performed with a Roche 480 thermal cycler. The primer sequences used are provided in Table S1.

### 2.4 Cell proliferation, apoptosis and colony formation assays

Cells were collected and seeded with required concentration, and then apoptosis and proliferation were assessed using TransDetect Annexin V‐EGFP/PI Cell Apoptosis Detection Kit (Transgene) and Cell Counting Kit‐8 (CCK‐8; Transgene) following the manufacturer's instructions, respectively. For colony formation assays, 1000 cells/well were plated onto six‐well plates, which were incubated at 37°C and 5% CO_2_ until colonies were formed. After 10‐15 days, colonies were fixed using 0.05% crystal violet in 4% paraformaldehyde and counted.

### 2.5 Reactive oxygen species detection

For reactive oxygen species (ROS) detection in cells, dihydroethidium (DHE) (Sigma, Saint Louis, MO, USA) staining for superoxide was performed. Cells were incubated with 10‐20 mmol/L DHE at 37°C for 60 min. Ethidium staining was visualized using inverted fluorescence microscope. The levels of cellular superoxide anion corresponded to red signal.

### 2.6 Measurement of PGC‐1α mRNA stability

FTO‐overexpressing cell lines, mutFTO‐overexpressing cell lines and control cell lines were cultured in six‐well plates. Then actinomycin D (Calbiochem, Burlington, MA, USA) was added to 8 μg/mL at 0, 3 and 6 hours before cell collection, followed by RNA extraction and real‐time PCR as described earlier.

### 2.7 Luciferase reporter and mutagenesis assays

The DNA fragments of PGC‐1α‐3′UTR containing the wild‐type m6A motifs as well as mutant motifs (m6A was replaced by C) were directly synthesized from GeneCreate (Wuhan, China). Wild‐type and mutant PGC‐1α‐3′UTR were inserted into downstream of firefly luciferase of pMIR‐GLO vector^38^ (pmirGLO Dual‐Luciferase miRNA Target Expression Vector; Promega, Madison, WI, USA). For dual‐luciferase reporter assay, 100 ng wild‐type or mutant PGC‐1α‐3′UTR, 200 ng pCMV3‐FTO (or pCMV3‐FTO‐Mut or pCMV3‐flag) were cotransfected into HEK‐293T cells in 24‐well plate. The relative luciferase activities were assessed 48 hours after transfection by Dual‐Luciferase Reporter Assay System (Promega). Each group was repeated in triplicate. The sequences of PGC‐1α‐3′UTR with wild‐type and mutant m6A sites were shown in Table S2.

### 2.8 Relative quantification of mitochondrial DNA content

Mitochondrial DNA content was measured by real‐time PCR as previously described.^38^, ^39^ Mitochondrial specific primers: mtMito‐F CACTTTCCACACAGACATCA; mtMito‐R TGGTTAGGCTGGTGTTAGGG. Nuclear‐specific primers: nuB2M‐F TGTTCCTGCTGGGTAGCTCT; nuB2M‐RCCTCCATGATGCTGCTTACA.

### 2.9 ATP measurements assay

Cells ATP measurements were obtained using Enhanced ATP Assay Kit (Beyotime, Shanghai, China) following the manufacturer's instructions.

### 2.10 Western blot

Cells were washed twice with ice‐cold PBS and ruptured with RIPA Lysis Buffer (#P0013B; Beyotime), PMSF, cocktail inhibitor and phosphatase inhibitor cocktail. Cell extracts were microcentrifuged for 15 minutes at 12 000 ***g*** and supernatants were collected. Cell lysates (15‐20 μL) were resolved by SDS‐PAGE and transferred onto PVDF membranes. Membranes were blocked for 1 hour with 5% non‐fat milk in TBST (Trisbuffered saline containing 0.1% Tween 20) and incubated overnight at 4°C with anti‐FTO antibody (ab124892; Abcam, Shanghai, China), anti‐β‐actin (3700S; Cell Signal Technology, Danvers, MA, USA), anti‐Vinculin (ab129002, Abcam), anti‐PGC‐1α (ab54481, Abcam). Membranes were washed 5 minutes with TBST for three times, incubated for 1 hour with required secondary antibodies conjugated to horseradish peroxidase and developed by chemiluminescent substrates.

### 2.11 In Vivo subcutaneous xenograft models

Five‐ to 6‐week‐old male athymic nude mice purchased by Charles River were used for the xenograft model. 769‐P cells stably expressing Ctrl, FTO and FTO‐mut were trypsinized and washed twice to thrice with standardized PBS, and then, 5 × 10^6^ cells in 100 μL of PBS was subcutaneously injected into the flanks of the mice (five mice per group). Mice were monitored twice every week for tumour growth, and tumour diameters were measured using a caliper. Tumour volume in mm^3^ was calculated using the formula: Tumour volume = (width^2^ × length)/2. Eight weeks after inoculation, mice were killed according to the policy for the humane treatment of tumour‐bearing mices. All animal studies were approved by Institutional Animal Care and Use Committee of Peking University.

### 2.12 Statistical analysis

Data in graphs are presented as mean ± SD or mean ± SEM. Differences between two groups or multiple groups were analysed by Student's *t* test and ANOVA, respectively. All statistical analyses were performed and *P* values were obtained using the GraphPad Prism software 6.0 or SPSS 20 (SPSS Inc., Chicago, IL, USA). *P* values <0.05 were considered significant.

## 3 RESULTS

### 3.1 FTO is down‐regulated in ccRCC and its expression is progressively lost during cancer progression

To explore the role of FTO in ccRCC progression, we first investigated the expression levels of FTO in a RCC sample cohort consisting of 35 pairs of primary ccRCC and adjacent normal tissues by qRT‐PCR, as shown in the Figure 1A, compared with the matched adjacent normal tissues, FTO was strongly down‐regulated in ccRCC tissues. Furthermore, an evident decreasing trend was observed across the early stage (I‐II), which also extended to late stage ccRCC (III‐IV) (Figure 1B). Consistently, FTO protein was reduced in a group of four pairs of ccRCC tissues compared with adjacent normal tissues as examined by Western blot (Figure 1C). To confirm the reduced FTO protein expression in a larger sample set, and correlate this to clinical phenotype, we performed immunohistochemical staining (IHC) on the FTO tissue array comprised of 25 patients. IHC showed that FTO was steadily expressed in normal kidney tissues but was declined in cancer counterpart and lost in the later stage (Figure 1D_1‐2_). To further assess the impact of FTO expression in clinical cases of ccRCC, we next analysed RNA sequencing data from over 500 patients with ccRCC from the Cancer Genome Atlas (TCGA). These data showed that low expression of FTO was markedly correlated with worse overall survival and disease‐free survival than patients whose tumours expressed relatively high levels of FTO (Figure 1E). Collectively, these results indicate that the FTO expression is frequently down‐regulated in ccRCC and associated with poor prognosis, suggesting that FTO may function as a tumour suppressor in ccRCC development.

Moreover, analyses of previously published gene expression datasets and TCGA database showed that FTO was significantly down‐regulated in various types of human cancer, such as breast, endometrial, uterine cervix cancer and bladder cancer (*P* value = 0.05) (Fig. S1A), and FTO low expression correlated with poor prognosis in human cancers, including endometrial cancer, lung cancer, rectum adenocarcinoma and pancreatic cancer (Fig. S1B), which further suggested that FTO may play an antioncogenic role in progression and development of various cancer types.

### 3.2 Ectopic expression of FTO inhibits cell growth, and induces apoptosis in ccRCC

**Figure 2:**
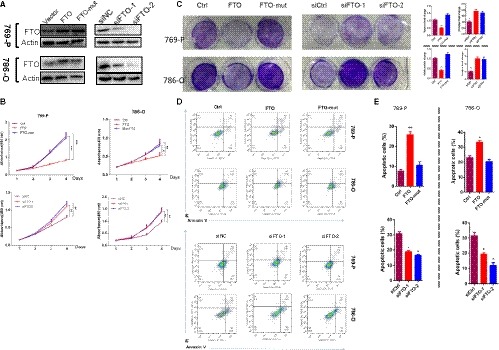
Ectopic expression of FTO inhibits cell growth, and induces apoptosis of clear cell renal cell carcinoma (ccRCC) in vitro. Western blotting showing forced expression and knockdown efficiency of FTO in 769‐P and 786‐O cells. Ectopic expression of FTO suppressed proliferation of ccRCC cells determined by CCK‐8 (B) and colony formation assays (C) while inducing cell apoptosis (D, E). Error bars represent the mean ± SEM of at least three independent experiments. *P < 0.05, **P < 0.01, ***P < 0.001

**Figure 3:**
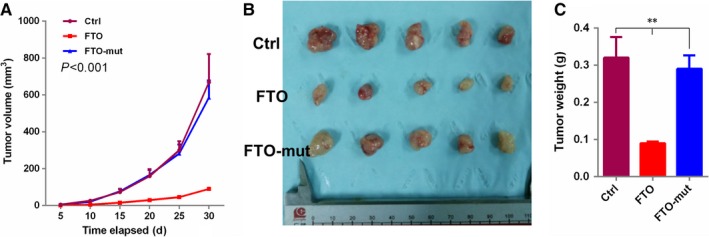
FTO significantly suppresses in vivo xenograft growth. (A) Subcutaneous tumour volume of 769‐P cell expressing a pCDH‐vector (n = 5), pCDH‐FTO (n = 5) or pCDH‐mutFTO (n = 5). The curve shows the mean ± SEM for each group, *P* < 0.001. (B) Photograph shows tumours generated from 769‐P cells transduced with FTO or mutFTO or vector lentivirus in (A) at the time of mouse euthanasia. (C) Quantification of tumour weight from xenograft mouse models. Data are presented as means ± SEM (n = 5). **P < 0.01

To evaluate the pathological role of FTO in ccRCC, both gain‐ and loss‐of‐function studies were performed in 786‐o and 769‐p cell lines. As shown in Figure 2A‐E, wild‐type FTO, but not the FTO mutant (two point mutations, H231A and D233A, which lead to disruption of the enzymatic activity of FTO^40^), reduced cell time‐dependent proliferation, anchorage‐independent growth and increased apoptosis; conversely, FTO knockdown resulted in an increase in cell proliferation and a reduce in apoptosis. Next, we established stable FTO overexpression cells using retroviral construct. FTO overexpression stable cells exhibited significantly suppressed cell proliferation in vivo xenograft growth (Figure 3A‐C), compared with the Vector control or FTO mutant (FTO‐mut) overexpression cells. These results demonstrated the anti‐carcinogenic role of FTO.

### 3.3 FTO regulates mitochondrial biogenesis and oxidative phosphorylation, while inducing oxidative stress at transcriptional level

**Figure 4:**
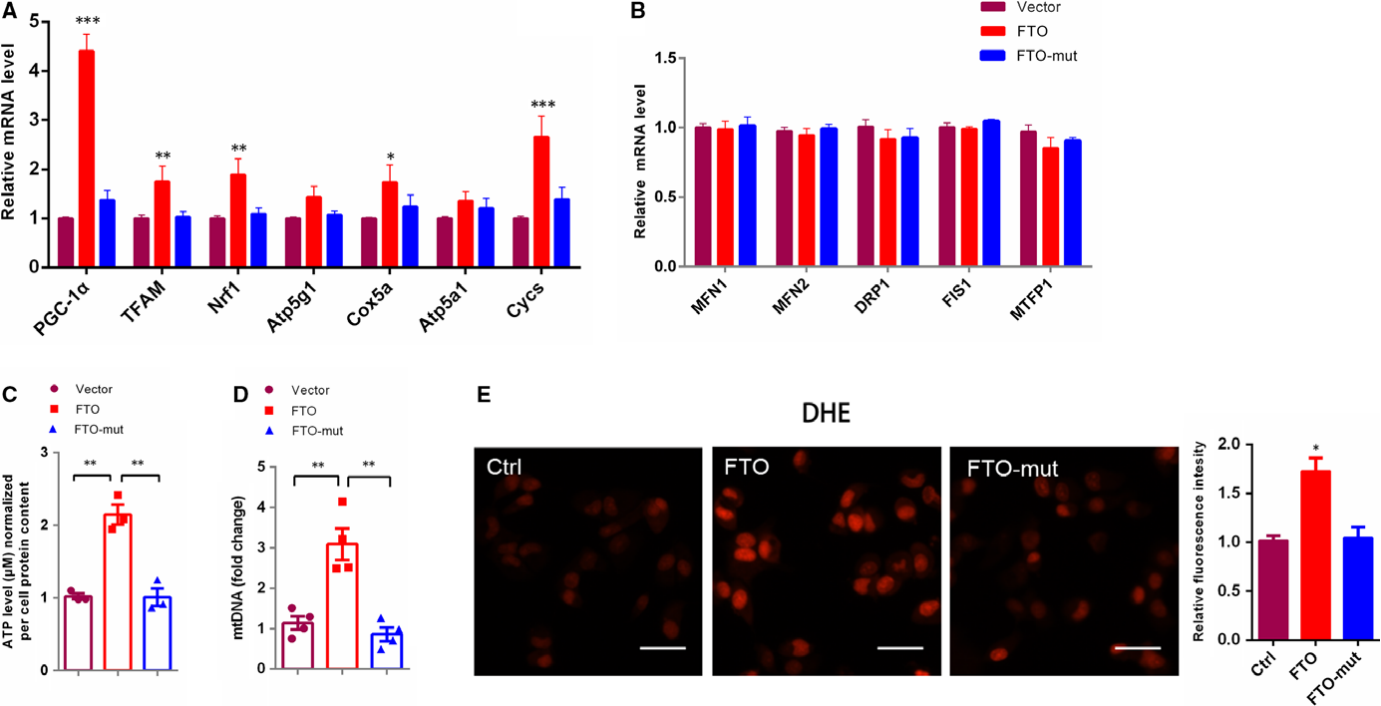
FTO regulates mitochondrial biogenesis and oxidative phosphorylation, while inducing oxidative stress at transcriptional level. (A, B) qPCR analysis of mitochondrial gene expression in 769‐P cells expressing vector, FTO or FTO‐mut construct. (C) qPCR of mitochondrial DNA (mtDNA) in 769‐P cells expressing FTO, FTO‐mut or vector. (D) Intracellular ATP levels in 769‐P cells expressing FTO, FTO‐mut or vector. (E) ROS levels in cells, as measured by the dihydroethidium (DHE) assay. Scale bars, 50 μm. Error bars indicate mean ± SEM, n = 3 or 4 for technical replicates. *<0.05, **<0.01, ***P < 0.001

Mitochondria act as a key hub of oxidative stress and apoptosis regulation on cell death in mammalian. Excessive mitochondria‐generated ROS can lead to oxidation of macromolecules and has been related to mtDNA mutations, ageing and cell death.^41^ Recent studies have demonstrated that meclofenamic acid, a highly selective inhibitor of FTO, reduce ROS accumulation and apoptosis,^42^ and that FTO regulates mitochondrial function.^35^, ^36^ We have been suggested that FTO might act as a pivotal regulator of mitochondrial activity and oxidative stress. Quantitative PCR assays revealed specific up‐regulation of genes relate to mitochondrial biogenesis (PGC‐1α, NRF1 and TFAM) and oxidative phosphorylation (Cox5a, Atp5 g1, Atp5a1 [ATPsynth] and Cycs [CytC]) in FTO overexpression cells compared with control or FTO‐mut overexpression cells, while the expression of genes regulating mitochondrial fission and fusion does not change in FTO‐overexpressing cells (Figure 4A,B).

To test whether increased expression of genes correlated with mitochondria biogenesis and oxidative phosphorylation in ccRCC associated with increased mitochondria number, mitochondrial energy production and oxidative stress, we first measured mitochondria DNA content, ATP production and ROS (Figure 4C‐E). FTO‐up‐regulating cells showed an increased mitochondrial DNA content (Figure 4C), an elevated cell ATP levels (Figure 4D) and an elevate ROS byproducts (Figure 4E). Above data suggested that FTO‐induced mitochondrial biogenesis and oxidative phosphorylation were closely relevant to oxidative stress in ccRCC cells.

### 3.4 FTO demethylates PGC‐1α mRNA and its stability is required for the regulatory role of FTO in ccRCC

**Figure 5:**
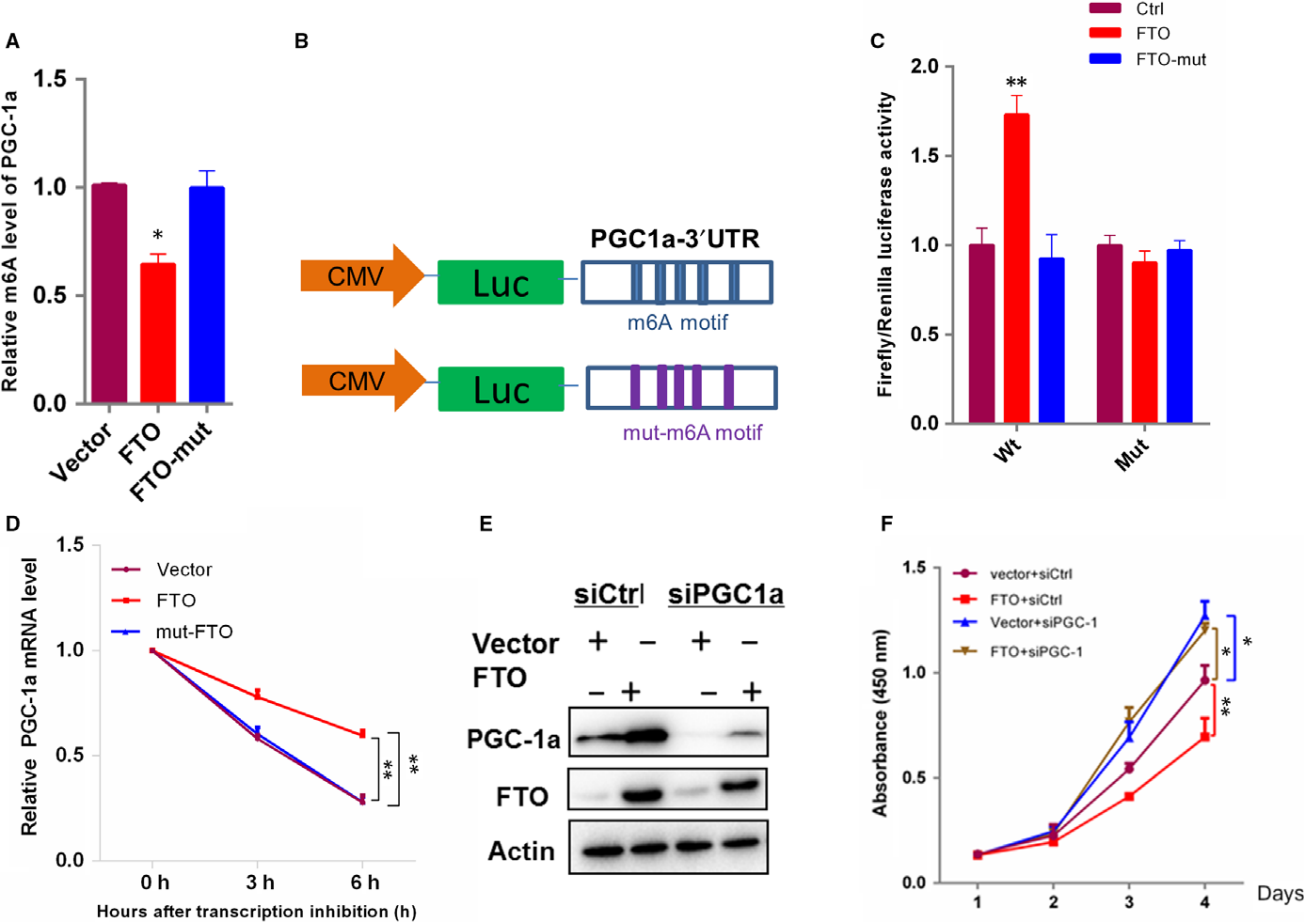
FTO demethylates PGC‐1α mRNA and its stability is required for the regulatory role of FTO in ccRCC. (A) Gene‐specific m6A qPCR analysis of m6A level in mRNA transcripts of PGC‐1α in 769‐P cells transduced with FTO,FTO‐Mut, or control vector (Ctrl). (B, C) Relative luciferase activity of pMIR‐GLO‐FTO‐3′ UTR with either wild‐type or mutant (A‐to‐C mutation) m6A sites after co‐transfection with FTO,FTO‐Mut or control vector (Ctrl) into HEK293T cell. Firefly luciferase activity was measured and normalized to Renilla luciferase activity. (D) The mRNA stability of PGC‐1α in 769‐p cells transfected with FTO,FTO‐Mut or control vector (Ctrl). Western blot of FTO and PGC‐1α (E) and cck8 assay (F) after treatment with PGC‐1α siRNAs in 769‐P cells with stable overexpression of FTO and empty vector. Error bars indicate mean ± SEM, n = 3 for technical replicates. *P < 0.05, **P < 0.01

PGC‐1α, as a central regulator of mitochondrial function, cannot be substituted for by the other family members^15^, ^16^ and is significantly up‐regulated by FTO expression (Figure 4A). Importantly, PGC‐1α inhibits ccRCC tumour growth by inducing oxidative stress.^17^ We reasoned that PGC‐1α might be a functionally important potential target gene of FTO in ccRCC. To prove it, we first performed gene‐specific m6A qPCR assays. The results demonstrated that overexpression of FTO reduced the m6A level of PGC‐1α mRNA (Figure 5A). To further elucidate the effect of the PGC‐1α mRNA m6A modifications for FTO‐mediated gene regulation, we performed PGC‐1α 3′‐UTR luciferase reporter and mutagenesis assays (Figure 5B). Mutation was at 5 m6A consensus sequences (RR (m6) ACH to RRCCH) within the PGC‐1α 3′‐UTR. As expected, luciferase activity of PGC‐1α 3′‐UTR luciferase reporter was significantly increased upon FTO overexpression, while mutations in the m6A sites rendered resistance to the effect of FTO overexpression (Figure 5C). Collectively, our data suggested that FTO‐mediated PGC‐1α up‐regulation depends on its catalytic activity of m6A demethylation. To further measure the role of m6A level reduces the stability of potential target transcripts of FTO, actinomycin D was used to inhibit global mRNA transcription. As demonstrated in Figure 5D, FTO significantly increased the PGC‐1α mRNA expression after inhibiting transcription, indicating that FTO enhanced PGC‐1α mRNA stability at least in part as a result of decreased m6A level. To further explore the role of the FTO‐PGC1α regulatory axis in ccRCC, we performed the PGC‐1α knockdown in FTO stably overexpressing 769‐P cells, and observed that FTO regulation of oxidative stress and cell proliferation could be largely reversed by PGC‐1α knockdown (Figure 5E,F). Together, our finding suggested that PGC‐1α is a functionally important target of FTO and the anti‐carcinogenic role of FTO is at least in part dependent on it.

### 3.5 PGC‐1α expression inhibits ccRCC tumour growth

**Figure 6:**
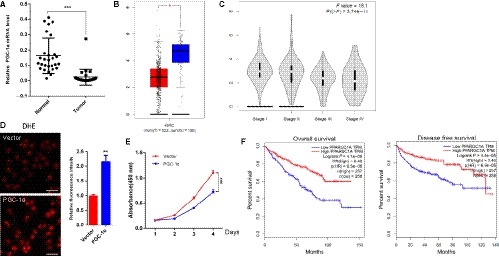
PGC‐1α expression inhibits tumour growth. (A) PGC‐1α down‐regulation was validated in 28 pairs of human ccRCC and their paired normal samples by qRT‐PCR. Error bars indicate mean ± SD, ***P < 0.001. (B, C) PGC‐1α down‐regulation was also found in TCGA ccRCC cohort. (D) ROS levels in cells expressing PGC‐1α or vector, as measured by the dihydroethidium (DHE) assay. Scale bars, 50 μm. (E) Forced expression of PGC‐1α suppressed proliferation of ccRCC cells determined by CCK‐8. (F) Kaplan‐Meier Curve for overall survival and disease free survival of ccRCC patients segregated by low (bottom quartile) or high expression of PGC‐1α. Log‐rank test *P* < 0.0001. Error bars indicate mean ± SEM, n = 3 for technical replicates. **P < 0.01

Given our results that ectopic FTO expression increased the expression of PGC‐1α, induction of oxidative stress and suppression of ccRCC growth, which led to our test whether ectopic expression of PGC‐1α can inhibit ccRCC growth. To support it, we first identified PGC‐1α mRNA was down‐regulation in 28 pairs of ccRCC (Figure 6A). Consistent with our data, PGC‐1α was also significantly down‐regulated in Gene Expression Profiling Interactive Analysis (GEPIA) database derived from TGCA RNA‐Seq dataset of 523 ccRCC and 72 normal samples (Figure 6B). More specially, Figure 6C showed that low PGC‐1α expression is associated with more severe tumours. Next, we validated that PGC‐1α restored expression induced oxidative stress (Figure 6D) and inhibited the ccRCC cell lines growth in vitro (Figure 6E). These results suggested that low PGC‐1α expression may be associated with worse patient survival. We referred to GEPIA to test whether there is an association between PGC‐1α expression and overall survival or disease‐free survival. PGC‐1α down‐regulation, as expected, was related to worse disease progression and reduced overall survival in 257 and 258 patients with ccRCC, respectively (Figure 6F).

## 4 DISCUSSION

The data presented here show that FTO, a member of the AlkB subfamily of FeII/α‐ketoglutarate‐dependent dioxygenases and the first identified m6A demethylase, act as a tumour suppressor in ccRCC. In vitro, we demonstrate that overexpression of FTO (not FTO‐mut) significantly inhibits the proliferation of human VHL‐deficient ccRCC cell lines, while increasing the apoptosis of the cells, the opposite is true when endogenous expression of FTO is silenced. In keeping with our results, prior study also suggested that meclofenamic acid (a highly selective inhibitor of FTO) reduces ROS accumulation and apoptosis.^42^ In vivo, we demonstrate that forced expression of FTO significantly inhibits tumour growth in xenograft nude mice model.

Furthermore, we demonstrate that forced expression of FTO in VHL‐deficient ccRCC cell lines facilitates mitochondria function depending on its m6A demethylase activity as mutations in the FTO catalytic domain significantly diminish the function of FTO. Remarkably, the increases in mitochondria biogenesis and oxidative phosphorylation upon expression of FTO are correlated with stimulation of oxidative stress in VHL‐deficient ccRCC cell lines. Specific mechanically, we demonstrate that FTO post‐transcriptionally demethylates and stabilizes PGC‐1α mRNA. Our finding was in line with previous studies^17^ that show the effect to facilitate mitochondria biogenesis and the anti‐tumorigenic role of PGC‐1α in VHL‐deficient ccRCC. Therefore, we revealed a novel FTO‐PGC‐1α axis signalling in suppressing ccRCC cell growth. In the case of ccRCC, the induced production of ROS upon FTO‐PGC‐1α pathway is inadequately detoxified, leading to oxidative stress. Whether the antioxidant response dictates outcome to enhanced mitochondrial biogenesis downstream of the pathway activation will require further exploration. Further studies are also required to determine whether ALKBH5, the other known m6A demethylase, can regulate the mitochondrial function and whether ALKBH5 have different or redundant roles compared to FTO.

**Figure 7:**
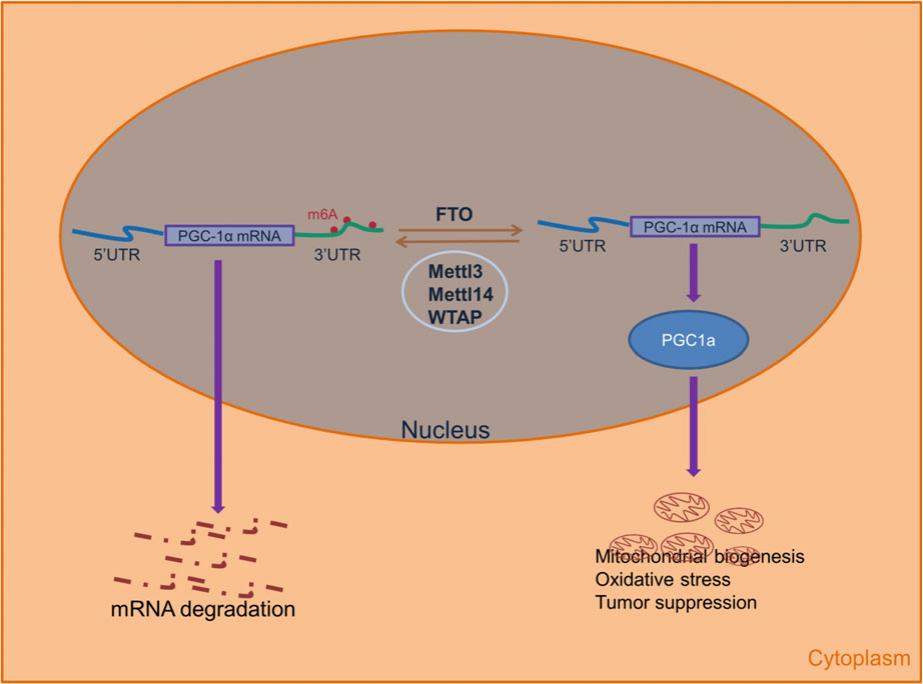
A model illustrating FTO‐mediated PGC‐1α expression and mitochondrial activity in ccRCC cells. FTO mediates demethylation of adenosine residues in the 3′‐UTR of PGC‐1α mRNA, leading to increased PGC‐1α mRNA stability and protein expression, and increased mitochondrial biogenesis, oxidative stress and tumour suppression

FTO, as m6A “erasers”, has also been reported to have pro‐tumorigenic function in acute myeloid leukaemia and glioblastoma,^34^, ^37^ but we show that FTO plays a critical anti‐tumorigenic role in ccRCC. The reasons for this heterogeneous role remain unclear. One possibility is that m6A modification is dynamic and tissue‐specific process, thus performing distinct genetic programs in variant cancer types. A better understanding of the role of FTO (or m6A) in variant tumour types and at different stages of tumorigenesis will be significant in testing whether this pathway will be useful to therapeutic intervention. In addition, several groups have found that m6A regulates circRNA translation efficiency,^43^ microRNA biogenesis^44^ and autophagy.^45^ These findings further provide profound insight into the bio‐relevant roles of m6A modification and contribute to understand the biological basis of cancer. Here, as shown in Figure 7, we present compelling evidence demonstrating that FTO plays a critical anti‐tumorigenic role in ccRCC. Restored expression of FTO, through reducing m6A levels in mRNA transcripts of its critical target gene PGC‐1α, increases mitochondrial content, ROS production and oxidative damage, with the most important effect of repressed tumour growth. The induction of novel FTO‐PGC‐1α axis may present an opportunity to enhance efficacy and improve the treatment of ccRCC.

## ACKNOWLEDGEMENTS

The authors thank Zeguang Lin for providing material support. The authors thank Xiaoqiang Guo, Qian Ma, Bo Yang, Wenlin Chang, Zeng Zhang, Liqiang Yang for providing useful technical advice and helpful comments on this manuscript. The authors also thank mice sacrificed for this study. This work was supported by grants from the Guangdong Key Laboratory of Male Reproductive Medicine and Genetics (2017B030314074), the Shenzhen Project of Science and Technology (JCYJ20170413100245260) and the “San‐ming” Project of Medicine in Shenzhen (SZSM201612066).

## COMPETING INTERESTS

The authors declare no competing interests.

## AUTHOR CONTRIBUTION

YTG, FTZ and CSZ conceived the project, designed and performed the research, analysed and interpreted data and wrote the manuscript. CSZ and CLZ performed statistical analysis and part of mice experiments. XML, XBH, LY, JFL, TFX, YWL provided assistance in some experiments and reviewing of the manuscript.
