# Supplementary figures and images for: N6‐methyladenosine demethylase FTO suppresses clear cell renal cell carcinoma through a novel FTO‐PGC‐1α signalling axis
Source: J Cell Mol Med. 2019 Jan 16;23(3):2163–73. doi: 10.1111/jcmm.14128 (PMC6378205; doi:10.1111/jcmm.14128)

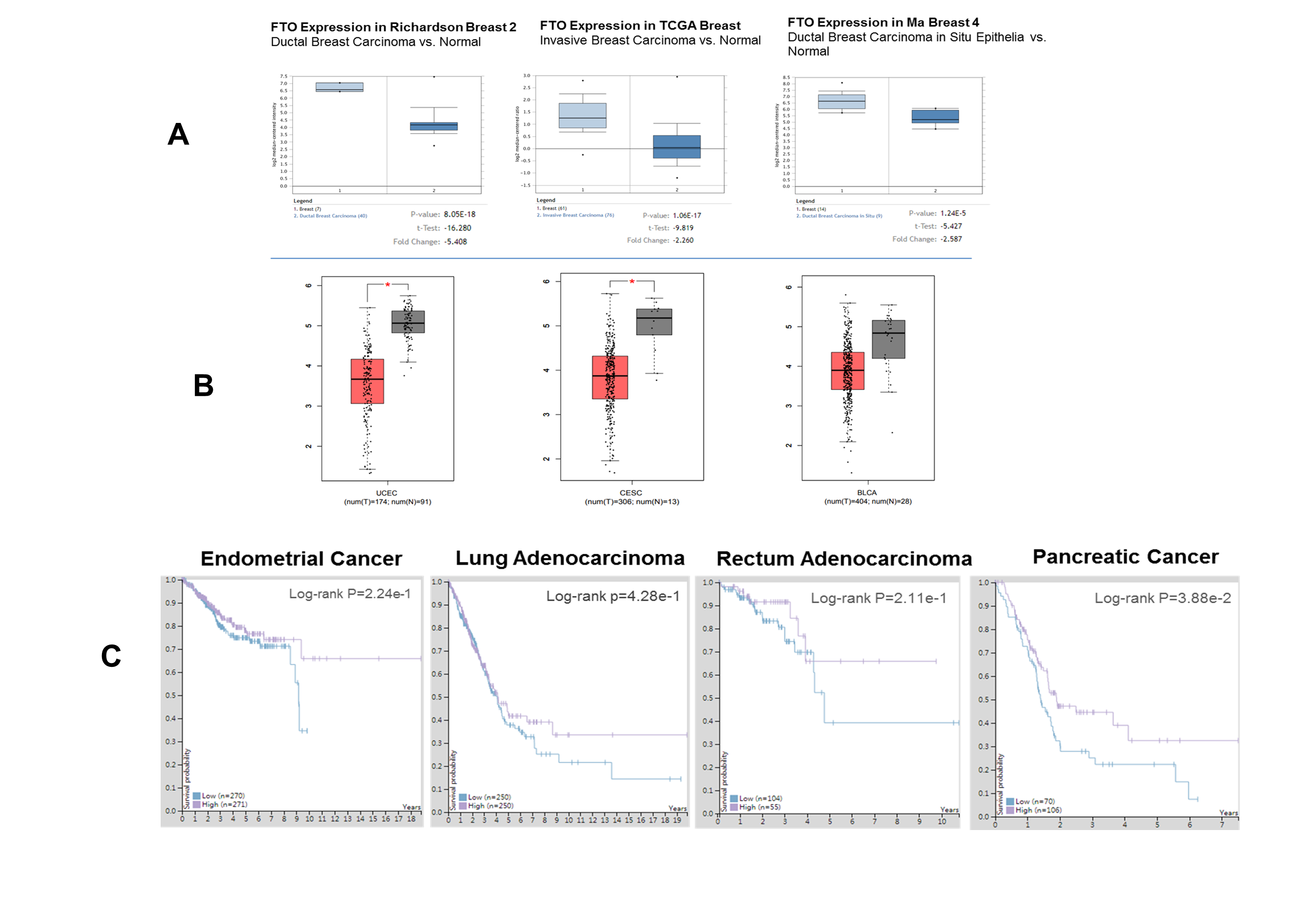

Supplement: Supplementary file 1 [file JCMM-23-2163-s001.tif]
