# Supplementary material for: N6‐methyladenosine demethylase FTO suppresses clear cell renal cell carcinoma through a novel FTO‐PGC‐1α signalling axis
Source: J Cell Mol Med. 2019 Jan 16;23(3):2163–73. doi: 10.1111/jcmm.14128 (PMC6378205; doi:10.1111/jcmm.14128)
Supplement: Supplementary file 2 [file JCMM-23-2163-s002.pdf]

Table S1

gene/name	primer seq			siRNA	seq
**hPgc1a-F**	GCTGCTGAAGAGGCAAGAGA			**siFTO1-sense**	GUGGCAGUGUACAGUUAUATT
**hPgc1a-R**	TGAAATGGTTTGCCCTTGCG			**siFTO1-anti**	UAUAACUGUACACUGCCACTT
**GAPDH-F**	GCATCCTGGGCTACACTGAG			**siFTO2-sense**	GGCAAUCGAUACAGAAAGUTT
**GAPDH-R**	GTCAAAGGTGGAGGAGTGGG			**siFTO2-anti**	ACUUUCUGUAUCGAUUGCCTT
**FTO-F**	GCATGGCTGCTTATTTCGGG			**siCtrl-sense**	UUCUCCGAACGUGUCACGUTT
**FTO-R**	GGATGCGAGATACCGGAGTG			**siCtrl-anti**	ACGUGACACGUUCGGAGAATT
**hNRF1-F**	AGGAACACGGAGTGACCCAA			**siPGC1a-1-sense**	CCAAGACUCUAGACAACUATT
**hNRF1-R**	TGCATGTGCTTCTATGGTAGC			**siPGC1a-1-anti**	UAGUUGUCUAGAGUCUUGGTT
**hTFAM-F**	ATCTCCGCGGCTCTTATTCC			**siPGC1a-2-sense**	CCAUAUUCCAGGUCAAGAUTT
**hTFAM-R**	CAATCACAACTGGAACCCGC			**siPGC1a-2-anti**	CCAUAUUCCAGGUCAAGAUTT
**hCox5a-F**	CCGTGGCTATCCAGTCAGTT			**siPGC1a-3-sense**	GCUCAGAGAAGCUUGCGCATT
**hCox5a-R**	ATGTTACCCAGCGAGCATCA			**siPGC1a-3-anti**	UGCGCAAGCUUCUCUGAGCTT
**hCox5b-F**	ATGGCTTCAAGGTTACTTCGC				
**hCox5b-R**	CCCTTTGGGGCCAGTACATT				
**hATP5a1-F**	TGCCCAGTTGGTGAAGAGAC				
**hATP5a1-R**	CTCCCATGGAACAGCCAGAG				
**hCycs-F**	ACTCTTACACAGCCGCCAAT				
**hCycs-R**	TAAGTCTGCCCTTTCTTCCTTC				
**hATP5g1-F**	TGATCCGCTGTTGTACCAGG				
**hATP5g1-R**	CTGGAGTGGGAAGTTGCTGT				
**hCox4i-F**	ACTACCCCATGCCAGAAGAG				
**hCox4i-R**	TCATTGGAGCGACGGTTCATC				
**MFN1-F**	GTGGTGTGGCACTTGCTGAA				
**MFN1-R**	ACTGCTGACTGCGAGATACAC				
**MFN2-F**	ATGCCTGTCACCAAGGTGTT				
**MFN2-R**	GAAGCGATCCAACTTGTGCC				
**FIS1-F**	GGAACAGCGGGATTACGTCT				
**FIS1-R**	CAACCCGCGGACGTACTTTA				
**MTFP1-F**	TGTGGTGGACACCTTTGTATG				
**MTFP1-R**	AGCACACACGCGGTTGA				
**DRP1-F**	TGTGCAAAGCAGTTTGCCTG				
**DRP1-R**	CTTGGAGGACTATGGCAGCA				
**HPRT-F**	GGAGTCCTATTGACATCGCCA				
**HPRT-R**	CAACAATCCGCCCAAAGGGA				

Table S2

PGC-1α-3'UTR with wild-type m6A sites:

CATGTTCCCTAGCTGAGGATGACAGAGGGATGGCGAATACCTCATGGGACAGCGCGTCCTTCCCTAAAGACTATT
GCAAGTCATACTTAGGAATTTCTCCTACTTTACACTCTCTGTACAAAAACAAAACAAAACAACAACAATACAACA
AGAACAACAACAACAATAACAACAATGGTTTACATGAACACAGCTGCTGAAGAGGCAAGAGACAGAATGATATC
CAGTAAGCACATGTTTATTCATGGGTGTCAGCTTTGCTTTTCCTGGAGTCTCTTGGTGATGGAGTGTGCGTGTGTGC
ATGTATGTGTGTGTGTATGTATGTGTGTGGTGTGTGTGCTTGGTTTAGGGGAAGTATGTGTGGGTACATGTGAGGA
CTGGGGGCACCTGACCAGAATGCGCA

PGC-1α-3'UTR with mutant m6A sites:

CATGTTCCCTAGCTGAGGATGACAGAGGGATGGCGAATACCTCATGGGCCAGCGCGTCCTTCCCTAAAGACTATTG
CAAGTCATACTTAGGAATTTCTCCTACTTTACACTCTCTGTACAAAACCAAACCAAACCAACAACAATACAACAAG
AACAACAACAACAATAACAACAATGGTTTACATGAACACAGCTGCTGAAGAGGCAAGAGACAGAATGATATCCA
GTAAGCACATGTTTATTCATGGGTGTCAGCTTTGCTTTTCCTGGAGTCTCTTGGTGATGGAGTGTGCGTGTGTGCAT
GTATGTGTGTGTGTATGTATGTGTGTGGTGTGTGTGCTTGGTTTAGGGGAAGTATGTGTGGGTACATGTGAGGCCT
GGGGGCACCTGACCAGAATGCGCA
